# Long-Term Health-Related Quality of Life following Acute Type A Aortic Dissection with a Focus on Male–Female Differences: A Cross Sectional Study

**DOI:** 10.3390/jcm13082265

**Published:** 2024-04-13

**Authors:** Frederike Meccanici, Carlijn G. E. Thijssen, Arjen L. Gökalp, Annemijn W. Bom, Guillaume S. C. Geuzebroek, Joost F. ter Woorst, Roland R. J. van Kimmenade, Marco C. Post, Johanna J. M. Takkenberg, Jolien W. Roos-Hesselink

**Affiliations:** 1 Department of Cardiology, Erasmus MC, 3015 GD Rotterdam, The Netherlands; 2 Department of Cardiology, Radboud University Medical Center, 6525 GA Nijmegen, The Netherlands; 3 Department of Cardiothoracic Surgery, Erasmus MC, 3015 GD Rotterdam, The Netherlands; 4 Department of Cardiothoracic Surgery, Radboud University Medical Center, 6525 GA Nijmegen, The Netherlands; 5 Department of Cardiothoracic Surgery, Catharina Ziekenhuis Eindhoven, 5623 EJ Eindhoven, The Netherlands; 6 Department of Cardiology, St. Antonius Ziekenhuis, 3435 CM Nieuwegein, The Netherlands; 7 Department of Cardiology, University Medical Center Utrecht, 3584 CX Utrecht, The Netherlands

**Keywords:** acute type A aortic dissection, health-related quality of life, outcomes, cross-sectional, sex and gender

## Abstract

**Objectives**: Acute type A aortic dissection (ATAAD) is a life-threatening cardiovascular emergency, of which the long-term impact on health-related quality of life (HRQoL) and male–female-specific insights remain inadequately clarified. **Methods**: Consecutive adult ATAAD patients who underwent surgery were retrospectively included between 2007 and 2017 in four referral centers in the Netherlands, and baseline data were collected. The 36-Item Short-Form (SF-36) Health Survey was sent to all survivors between 2019 and 2021 and compared to validated SF-36 scores of the Dutch general population stratified by age group and sex. **Results**: In total, 324/555 surviving patients returned the SF-36 questionnaire (response rate 58%), of which 40.0% were female; the median follow-up was 6.5 years (range: 1.7–13.9, IQR: 4.0–9.4) after surgery for ATAAD. In comparison to the general population, ATAAD patients scored significantly lower on 6/8 SF-36 subdomains and higher on bodily pain. Differences in HRQoL domains compared to the sex-matched data were largely comparable between sexes, apart from bodily pain. In the age-matched subgroups impaired HRQoL was most pronounced in younger patients aged 41–60 (5/8 impaired domains). Female ATAAD patients scored significantly worse on 5/8 SF-36 subdomains and the physical component summary (PCS) scores than male patients. Age at ATAAD, female sex, hypertension, COPD, and prior thoracic aortic aneurysm were associated with worse PCS scores. **Conclusions**: Long-term HRQoL was impaired in both male and female ATAAD patients when compared to the general population. Further studies on the nature of this impairment and on interventions to improve HRQoL after ATAAD are clearly warranted, with special attention to females and younger patients.

## 1. Introduction

Acute type A aortic dissection (ATAAD) is an urgent cardiovascular event, requiring immediate diagnosis and emergency surgery. Although early mortality has significantly decreased over time for ATAAD, the in-hospital mortality is still around 20% [1,2]. The recovery process after ATAAD is often challenging: postoperative complications such as bleeding, stroke, and renal failure [3] can prolong hospitalization and impact health-related quality of life (HRQoL). Also, patients require lifelong imaging follow-up, and reintervention on the aorta may be warranted [4]. All these factors might cause fear and influence the performance of normal daily activities significantly, which raises the question whether more personalized attention for patients’ mental and physical wellbeing during clinical follow-up visits after ATAAD is required.

Recent literature has explored male–female differences in clinical presentation and management for ATAAD [5], yet a nuanced examination of how male and female patients perceive and experience HRQoL following ATAAD remains largely unexplored [6]. HRQoL has proven to be different for males and females in the general population as well as in patients with thoracic aortic disease [7,8], highlighting the need for a sex-specific evaluation of long-term HRQoL. Through examining male–female differences in various dimensions of HRQoL, including physical, emotional, and social well-being, this research can provide implications for tailored post-operative care strategies, risk stratification protocols, and patient-centered interventions to improve HRQoL.

Therefore, the primary objective was to investigate long-term HRQoL in adult ATAAD survivors in a large multicenter cross-sectional study in comparison with the Dutch general population. The secondary objectives were to examine male–female differences in HRQoL for the ATAAD population and to explore associations with patient and surgical characteristics and the physical and mental component summary scores.

## 2. Materials and Methods

### 2.1. Study Design and Study Population

Based on a multicenter retrospective cohort, a cross-sectional survey study was performed following the STROBE guidelines for cross-sectional studies [9]. All consecutive patients (≥18 years old) who presented with ATAAD between the 1 January 2007 and the 31 December 2017 in 4 tertiary referral centers in the Netherlands (the Erasmus University Medical Center in Rotterdam, the Catharina Medical Center in Eindhoven, Radboud University Medical Center in Nijmegen and St. Antonius Hospital in Nieuwegein) were eligible for inclusion. Exclusion criteria included: asymptomatic chronic type A aortic dissection, and iatrogenic or traumatic dissection. This multicenter study was designed, conducted, and controlled complying local and international good clinical practice guidelines and was approved by the local medical ethics committees with a waiver for informed consent for the retrospective data collection of all presenting ATAAD patients (MEC-2018-1535). Written informed consent was obtained from all surviving ATAAD patients for the collection of the HRQoL questionnaires.

### 2.2. Data Collection

Eligible patients were identified with the institutional aortic surgery databases and the hospitals’ diagnosis registration systems. Additionally, the files of all patients with diagnostic treatment codes (DBCs) pertaining to any thoracic aortic disease were checked manually to ensure the comprehensiveness of the patient selection.

All study data were documented in an anonymized standardized case report form using OpenClinica (OpenClinica, LLC, version 3.6, Needham, MA, USA). The patient files were used to collect data on demographic, clinical presentation, and treatment characteristics. All patient characteristics at baseline with their definitions are shown in Appendix SI. Prior to sending the questionnaire, a mortality check was performed in the municipal data registry. In the period from July 2019 to February 2021, the 36-Item Short-Form Health Survey (SF-36) [10,11,12] including an informed consent form on paper was sent to all ATAAD survivors. To improve the response rate, all patients who had not returned the questionnaire were contacted by telephone. After exhausting all the aforementioned attempts to elicit a response, the patient was classified as a ‘non-responder’.

### 2.3. HRQoL Questionnaire

The SF-36 questionnaire, comprising 36 items, is a widely employed tool for assessing HRQoL. It encompasses eight domains, namely: Physical Functioning (PF), Role limitations due to the Physical health problems (RP), Bodily Pain (BP), General Health perceptions (GH), Vitality (VT), Social Functioning (SF), Role limitations due to Emotional problems (RE), and general Mental Health (MH) (psychological distress) [7,12]. From these eight subdomains, two higher-ordered clusters are calculated: the Physical Component Summary (PCS) and the Mental Component Summary (MCS). The PCS and MCS are calculated by calculating the z-scores of each domain, followed by aggregation of scale scores and transformation of summary scores (T-scores). The first four domains (PF, RP, BP, GH) correlate most highly with the PCS, while the last four domains (VT, SF, RE, MH) are strongly correlated with the MCS [13]. The score range for all subdomains of SF-36 is 0–100, and for the PCS and MCS the mean score is 50 with a standard deviation of 10, with higher scores indicating better HRQoL.

Previous studies have utilized the SF-36 questionnaire as tool to examine HRQoL in patients with type A aortic dissection [6]. The SF-36 has been translated, validated, and normed in the Dutch population, as well as for the total population, and stratified by sex and age groups, which can be considered as normative data [7]. In order to compare the study data with the Dutch general population, the age categories used by Aaronson et al. [7] were applied to this study: age 16–40 years, 41–60 years, 61–70 years, and more than 70 years old.

### 2.4. Statistical Analysis

Data were analyzed using the statistical and computing program *R* (R Foundation for Statistical Computing, Vienna, Austria. Version 4.2.1). Continuous data were presented as mean and standard deviation (SD) when normally distributed and as median with interquartile range (IQR) when skewed. Normality was checked visually with histograms and tested with use of the Shapiro–Wilk test. Males and females were compared with an unpaired Student’s *t*-test when normally distributed, and a Mann–Whitney U test was used when data were not normally distributed. Categorical data were presented as counts and frequencies, and males and females were compared with χ2 test or Fisher’s exact test, as appropriate.

Furthermore, two subgroup comparisons were made with the Dutch normative data: (1) sex-matched, and (2) age-matched (per age category as reported by Aaronson et al. [7]). In the comparison with the normative data, means and standard deviations were reported and a one-sample Student’s *t*-test was used.

Associations with the PCS and the MCS were explored using univariable linear regression analyses with follow-up time, age at ATAAD, sex, comorbidities, and concomitant aortic valve and aortic arch surgery at ATAAD repair based on previous research and clinical relevance [6] in a complete case analysis. Multivariable analyses were performed adjusting for age and sex, and interactions with sex and the independent variables were checked. In an additional analysis, the association between postoperative cerebrovascular accidents and the PCS and MCS were explored in a univariable linear regression analysis.

To investigate non-responder and survival bias, the patient characteristics of responders were compared with non-responders of the total cohort using the aforementioned descriptive statistics for continuous and categorical data.

If the participant failed to fill in any data for one or more questions in a subdomain, the entire subdomain was regarded as missing. As a result, patients who had any incomplete subdomains would also have a missing end score of the PCS and MCS. If the participant did not provide the completion date for the questionnaire, the study center’s median date was used as an estimate.

A two-sided *p*-value of <0.05 was considered statistically significant.

## 3. Results

**Figure 1:**
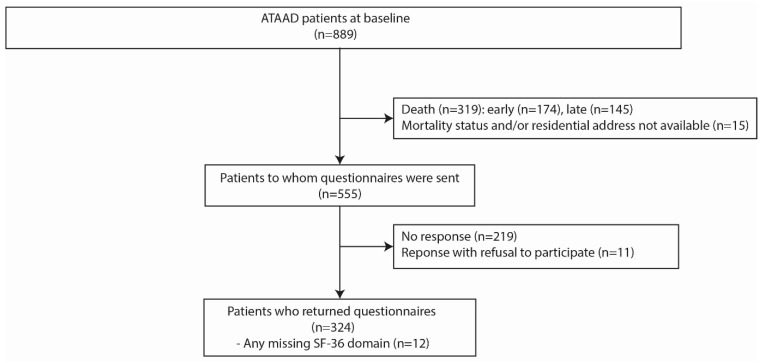
Flowchart of acute type A aortic dissection (ATAAD) patients.

In Figure 1, the flowchart of the patient selection is shown. The questionnaires were sent to 555 ATAAD survivors, of which 324 patients (40.0% females) completed the questionnaire and provided written informed consent, resulting in a response rate of 58.4%. In Table 1, the patient and surgical characteristics of all patients who completed the questionnaire (*n* = 324) are depicted stratified by sex. All ATAAD patients included in the present study had received surgical treatment at presentation. During admission or within 30 days after ATAAD surgery, cerebrovascular accidents were observed in 25 patients (7.9%, *n* = 8 females, *n* = 17 males) and transient ischemic attack in 2 patients (0.6%, *n* = 2 females). No significant male–female differences were observed in postoperative CVA/TIA incidence.

**Figure 2:**
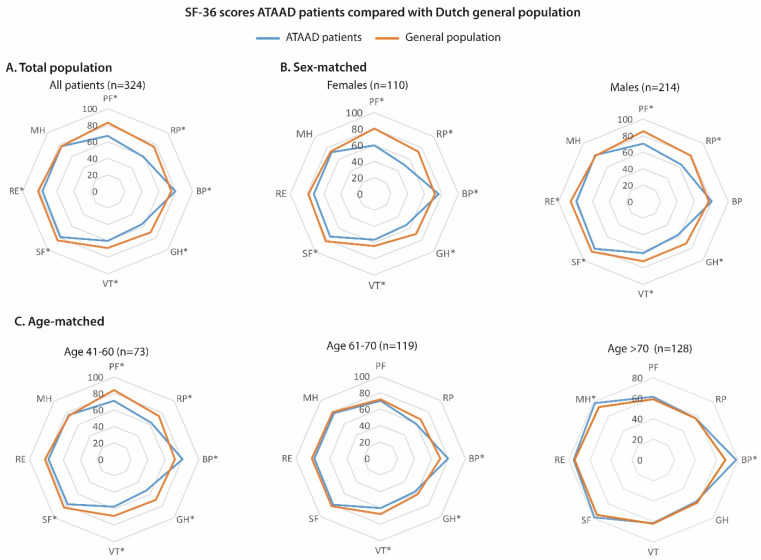
SF-36 scores of ATAAD patients compared with the Dutch general population. SF-36 subdomain scores are presented as the mean in order to compare the data with the general population data, which were reported as the mean. Three patients were in the age range of 16–40 years; therefore, these patients were not included in the age-matched analysis. * = *p*-value < 0.05 in one-sample Student’s *t*-test compared with the general population. ATAAD = Acute type A aortic dissection, SF-36 = 36-Item Short-Form Health Survey, PF = physical functioning, RP = role physical, BP = bodily pain, GH = general health, VT = vitality, SF = social functioning, RE = role emotional, MH = mental health.

In Figure 2, mean SF-36 subdomain scores are compared with the general Dutch population for the total study population (panel A), matched by sex (panel B) and matched by age (panel C). The crude data for these analyses are shown in Appendices SII and SIII. On 6/8 SF-36 subdomains, ATAAD patients scored significantly lower than the general population, whereas ATAAD patients scored higher for bodily pain (Figure 2, panel A). In the comparison with the sex-matched general population, both males and females scored significantly worse on physical functioning, role physical, general health, vitality, and social functioning, and the absolute differences with the general population seem greater for females on the physical functioning and role physical domain (Figure 2, panel B). Females scored significantly higher for bodily pain and males scored significantly lower for role emotional, when compared to the sex-matched data. In the age-matched subgroups, impaired HRQoL was especially observed in the youngest age category (41–60 years), with 5/8 domains significantly lower than the age-matched general population, compared to 2/8 impaired domains in the age group of 61–70 years and none in the age group of >70 years (Figure 2, panel C).

Table 2 shows the SF-36 sub-domain scores and the PCS and MCS for the total study population and stratified by sex. Female patients scored significantly lower on 5/8 subdomains and on the PCS when compared to male patients.

**Figure 3:**
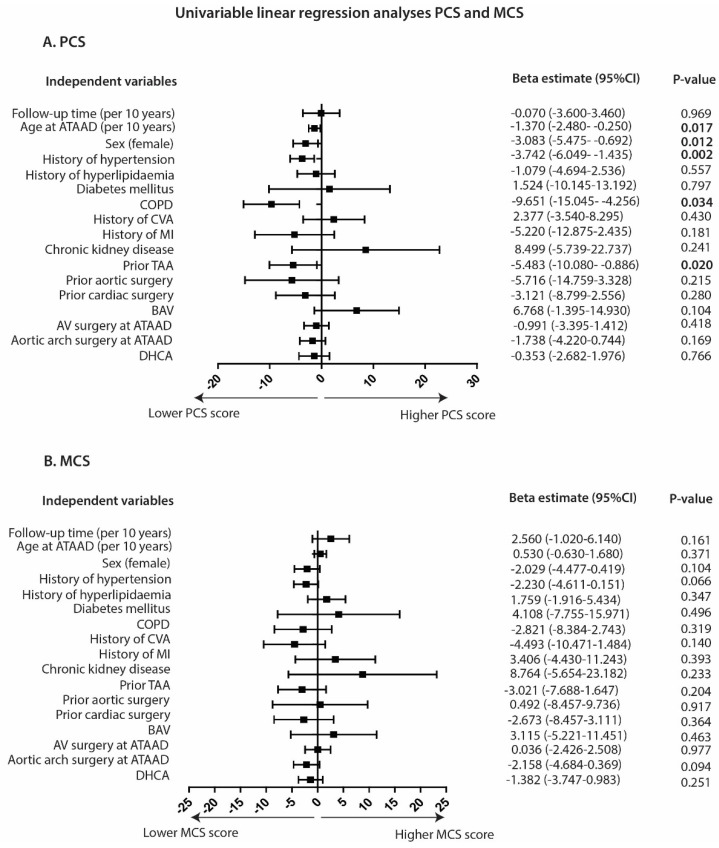
Associations with Physical Component Summary and Mental Component Summary scores. Beta coefficients and corresponding 95% CI are shown. Interpretation for beta coefficients: if the beta coefficient is positive, for every unit increase in the predictor variable, the outcome variable (PCS or MCS score) will increase by the beta coefficient value. *p*-values < 0.05 are depicted in bold. ATAAD = acute type A aortic dissection; PCS = physical component summary; MCS = mental component summary; COPD = chronic obstructive pulmonary disease; CVA = cerebrovascular accident; MI = myocardial infarction; TAA = thoracic aortic aneurysm; BAV = bicuspid aortic valve; AV = aortic valve; DHCA = deep hypothermic cardiac arrest.

Figure 3 shows the univariable analyses of patient and surgical characteristics with the PCS and the MCS for the total study population. As depicted in Appendix SIV, after correction for age and sex, the following variables remained significantly associated with lower PCS scores: history of hypertension (*p* = 0.003), COPD (*p* = 0.012), and prior thoracic aortic aneurysm (*p* = 0.043). For the MCS, no significant associations were found in univariable (Figure 3) and multivariable analysis (Appendix SIV). No significant interactions with sex and the independent variables were observed for both the PCS and MCS. Postoperative cerebrovascular accidents were not significantly associated with the PCS (beta estimate: −3.420 (95% CI −7.674–0.835), *p* = 0.115), nor with the MCS (beta estimate: 0.073 (95% CI −4.282–4.428), *p* = 0.974.

In Table 3, responders (*n* = 324) are compared with non-responders (*n* = 231), showing a higher proportion of smoking in the non-responders (82.4 vs. 62.9, *p* = 0.001) and a lower proportion of concomitant aortic valve surgery (52.5 vs. 66.5, *p* = 0.002) when compared with responders.

## 4. Discussion

In an era of advanced medical care and surgical techniques, patient-centered outcomes become more important. This is certainly true for patients with acute type A aortic dissection (ATAAD), for whom, historically, the most important outcome was survival. Through increased awareness of health-related quality of life (HRQoL) and potential sex differences, the current study will enhance a more personalized approach post-ATAAD. Female patients were older at surgery for ATAAD and at completion of the questionnaire and scored worse on 5/8 SF-36 subdomains when compared to male patients. Also, HRQoL was impaired in ATAAD patients when compared to the general population, especially in the younger age groups. In an explorative analysis, age at ATAAD, female sex, hypertension, COPD, and prior thoracic aortic aneurysm were associated with impaired physical component summary scores (PCS), whereas no significant associations with the mental component summary scores (MCS) were observed.

Overall, HRQoL in the study population was impaired when compared to the general population. Other literature comparing the HRQoL of ATAAD patients with age-adjusted normative data is conflicting: some studies show decreased HRQoL [14,15,16,17], while others show estimates comparable with the general population [18,19,20]. Patient age and follow-up duration, together with the sample size and statistical power in these studies, seem important factors to take into account. When measured longitudinally, Endlich and colleagues observed that HRQoL decreased during follow-up after ATAAD, especially concerning the mental component summary score in younger patients [15]. In a study investigating long-term HRQoL after cardiac surgery in general, a decay in HRQoL over time was more pronounced in older patients for 3/8 subdomains [21]. In the current study, measuring HRQoL at one moment during follow-up, the follow-up duration was not associated with either the physical or mental component summary score in linear regression.

Published evidence on HRQoL after ATAAD, unfortunately shows little attention to male–female differences [6]. The current study revealed that male ATAAD patients had higher scores than their female counterparts on 5/8 subdomains, reflecting a worse HRQoL in female ATAAD patients. However, this trend was also seen in the general Dutch population, where females had lower scores on even 7/8 subdomains [7]. The absolute difference with the general population seemed more pronounced in female patients on the physical domains, and female sex was associated with worse physical component summary scores. In a study on HRQoL in thoracic aortic disease patients, the physical domains also seemed more severely impaired in female patients compared to the sex-matched general population, than in male patients [8]. Of note, a study on physical activity after ATAAD found female sex to be an independent predictor for reduced physical activity [22]. Therefore, physical health warrants specific attention, and qualitative studies might explore the nature of this physical inactivity in female ATAAD patients.

Additionally, it is crucial to underscore the need to assess perceived physical impairments and mental health separately in survivors of ATAAD. With regard to physical status, in this cohort older age was associated with lower PCS scores, as described earlier [14,15,23]. Nevertheless, when compared with age-matched counterparts of the general population, comparable estimates on the physical domains for the age group >70 were observed, while worse scores were observed for the patients aged 41–60 years and 61–70 years. It could be possible that only the healthy elderly with little comorbidities have undergone surgery for ATAAD. These results underline the satisfactory HRQoL results for older patients after ATAAD surgery as reported [16,24].

A physically active lifestyle including mild to moderate exercise should be promoted in all adults but especially in ATAAD survivors [25], as it might improve their overall cardiovascular health by reducing blood pressure, maintaining a healthy body weight, and improving mental health [26]. Interestingly, a history of hypertension, prior thoracic aortic aneurysm, and COPD were associated with lower PCS scores, factors which have not been investigated in previous studies [6]. These conditions could reflect a poor cardiorespiratory condition with limited physical activity in these patients. It seems important to offer patient-tailored programs for different age categories, as the daily activities and expectation of physical health of a 60 year old differ greatly from those of an 80 year old. Also, patients could benefit from sex-specific programs, as females tend to be underrepresented in cardiac rehabilitation programs [27]. Future research should focus on the effect of clear exercise recommendations by healthcare providers and cardiac rehabilitation programs on the physical status of ATAAD surgery survivors.

Interestingly, ATAAD patients in general and female patients in particular had better scores for bodily pain when compared to the general population, indicating less pain and interference of pain with daily activities. In contrast, Jussli-Melchers et al. found that ATAAD patients scored worse on bodily pain [16]. Additionally, in a study on thoracic aortic disease, no significant differences were found in bodily pain scores when compared to the general population [8]. In long-term follow-up of adult congenital heart disease patients after surgical repair, generally better HRQoL scores were observed in comparison to normative data [28], reflecting a positive perception on life. Pain at ATAAD presentation is often described as ‘worst-ever pain’. One could speculate that when having experienced such intense pain, all the painful experiences afterwards are put into perspective.

ATAAD is a life-threatening traumatic event, accompanied with excessive pain and followed by emergency surgery—a sequelae of events triggering anxiety and psychological distress [14,25,29]. In this study, role emotional, social functioning, and vitality were impaired when compared to the general population, reflecting a significant impact on patients’ mental health. In the age-matched analysis, a pattern can be observed: with increasing age, mental-health-related domain scores increase relative to normative data. Endlich et al. observed a strong pattern of worse MCS in younger patients over time [15], indicating better coping with regard to mental wellbeing in older patients. Physicians should aim to provide guidance and understanding to younger patients, as the impact on their social environment and employment seems more significant than in older patients.

Interestingly, the subdomain of mental health, representing anxiety or depression, was not decreased compared to the general population. As the SF-36 is not designed to measure anxiety and psychological distress in depth, it might be underestimated in this study. It remains important for clinicians to screen patients after ATAAD for (unfounded) fears that might lead to inactivity and a decrease in HRQoL.

Surgical repair of ATAAD is usually a complex and lengthy procedure, which might impact HRQoL outcomes. A recent meta-analysis showed that females received less complex surgical repair than males and less frequently underwent aortic valve replacement at ATAAD presentation when compared to males [5]. There is an ongoing debate on the extent of the procedure in the acute phase of ATAAD, since reoperation on the distal aorta is common during follow-up. In one study on surgery of the proximal aorta, the use of DHCA was associated with worse HRQoL scores [20], which was not found in this study. Also, repair/replacement or arch repair were not significantly associated with PCS or MCS scores. Prospective HRQoL studies with measurements before and after intervention might provide more insight into a favorable approach with regard to HRQoL, although the effect of management on HRQoL is difficult to examine due to the inherent bias of observational studies.

## 5. Limitations

A few limitations need to be addressed. Since the study design is cross-sectional, no baseline or pre-dissection HRQoL were available, and HRQoL was assessed during follow-up at one time point, hampering a time-dependent analysis. In the current study, the response rate was 58%, comparable to other (online) questionnaires [30]. Some differences in baseline characteristics were observed between non-responders and responders, implicating non-responder bias. Furthermore, survival bias might be present: the relatively worse patients could have passed away preceding the follow-up moment. Also, the SF-36 might not capture all the disease-specific problems encountered by ATAAD survivors. Nonetheless, the SF-36 is a validated tool to measure HRQoL, which improves the comparison with other studies and the general population. As the baseline data collection was retrospective, we were unable to gather information on significant aspects related to HRQoL, such as cognitive abilities and psychiatric conditions.

## 6. Conclusions

In this large multicenter cross-sectional study, long-term HRQoL was clearly impaired in ATAAD patients when compared to the general population, and most pronounced in younger and female patients. Age- and sex-specific attention during follow-up after ATAAD might help improve HRQoL in these patients. Social aspects might be more important for younger patients than for older patients, while physical domains were especially affected in females and older patients. Due to the improvement in early mortality of ATAAD over the past years, ATAAD survivors will become lifelong patients, requiring a patient-tailored follow-up program and guidance. HRQoL could be improved by interventions such as clear lifestyle recommendations for patients and cardiac rehabilitation, with a more inclusive and holistic approach to patient care.

## Figures and Tables

**Table 1:** Patient and surgical characteristics of ATAAD patients who completed the SF-36 questionnaire.

	All Patients (*n* = 324)	Females (*n* = 110)	Males (*n* = 214)	*p*-Value	Missing %
Age at questionnaire (median [IQR])—years	68.0 [61.0–74.0]	70.5 [64.3–77.0]	67.0 [60.0–73.0]	**0.001**	0.3
Follow-up time—years (median [IQR], (range)	6.5 [4.0–9.4], (1.7–14.2)	6.2 [3.9–9.3], (1.7–13.9)	6.6 [4.2–9.4], (1.7–14.2)	0.386	0.0
**Patient demographics at ATAAD presentation**					
Age at ATAAD (mean ± SD)—years	61.0 ± 10.1	63.7 ± 10.3	59.7 ± 9.7	**0.001**	0.3
BSA (mean ± SD)—m^2^	2.0 ± 0.2	1.8 ± 0.16	2.1 ± 0.2	**<0.001**	23.5
History of hypertension (%)	150 (48.9)	59 (55.7)	91 (45.3)	0.107	6.2
History of hyperlipidemia (%)	37 (11.7)	13 (12.1)	24 (11.5)	1.000	2.8
Diabetes mellitus (%)	3 (0.9)	2 (1.9)	1 (0.5)	0.268 ^i^	2.2
COPD (%)	14 (4.4)	7 (6.4)	7 (3.3)	0.327	0.9
Current or past smoking ≥ 1 pack years	90 (62.9)	27 (56.2)	63 (66.3)	0.320	55.9
History of CVA or TIA (%)	14 (4.4)	6 (5.5)	8 (3.8)	0.686	0.9
History of MI (%)	7 (2.2)	3 (2.7)	4 (1.9)	0.695 ^i^	1.2
Chronic kidney disease (%)	3 (0.9)	1 (0.9)	2 (0.9)	1.000 ^i^	0.9
Prior TAA (%)	21 (6.6)	11 (10.1)	10 (4.7)	0.111	1.2
Prior aortic surgery (%)	5 (1.6)	0 (0.0)	5 (2.4)	0.170 ^i^	1.5
Prior cardiac surgery (%)	13 (4.1)	3 (2.7)	10 (4.8)	0.554 ^i^	1.2
Bicuspid aortic valve (%)	6 (2.1)	2 (2.0)	4 (2.2)	1.000 ^i^	11.4
Known connective tissue disease (%) * No; no genetic testing performed No; genetic testing performed but not found	13 (10.7) 67 (55.4) 41 (33.9)	3 (7.9) 20 (52.6) 15 (39.5)	10 (12.0) 47 (56.6) 26 (31.3)	0.602 ^i^	62.7
Surgical procedures **					
Aortic valve surgery Ascending aortic surgery Aortic arch surgery Descending aortic surgery	210 (66.5) 315 (97.2) 225 (70.8) 2 (0.6)	75 (70.8) 107 (97.3) 72 (67.9) 0 (0.0)	135 (64.3) 208 (97.2) 153 (72.2) 2 (0.9)	0.306 1.000 0.513 0.551 ^i^	2.5 0.0 1.9 0.6
DHCA (%)	124 (39.7)	45 (42.1)	79 (38.5)	0.630	3.7

Normally distributed continuous variables are expressed as mean ± SD, skewed continuous variables are expressed as median and 25th–75th percentile, and categorical values are expressed as percentages. For follow-up time, the 25th–75th percentile as well as the range are reported. *p*-values < 0.05 are depicted in bold. * Known connective tissue disease at ATAAD presentation: Marfan syndrome (*n* = 2), Loeys–Dietz syndrome (*n* = 1), ACTA2 mutation (*n* = 3), other (*n* = 5). ** All patients received surgical treatment at ATAAD presentation. ^i^ = Fisher’s exact test. IQR = interquartile range; BSA = body surface area; COPD = chronic obstructive pulmonary disease; CVA = cerebrovascular accident; DHCA = deep hypothermic circulatory arrest; MI = myocardial infarction; TAA = thoracic aortic aneurysm; TIA = transient ischemic attack.

**Table 2:** Health-related quality of life scores of the ATAAD study population for the 36-Item Short-Form Health Survey.

	All Patients (*n* = 324)	Females (*n* = 110)	Males (*n* = 214)	*p*-Value	Missing (%)
Physical Functioning	70.0 (53.8–85.0)	65.0 (42.5–80.0)	75.0 (60.0–90.0)	**<0.001**	1.2
Role Physical	75.0 (25.0–100.0)	50.0 (0.00–100.0)	75.0 (25.0–100.0)	**0.015**	2.2
Bodily Pain	90.0 (67.5–100.0)	82.5 (57.5–100.0)	90.0 (67.5–100.0)	0.142	0.3
General Health	60.0 (40.0–75.0)	52.5 (36.3–70.0)	60.0 (40.0–75.0)	0.103	0.0
Vitality	65.0 (45.0–80.0)	60.0 (40.0–75.0)	65.0 (45.0–80.0)	**0.035**	0.3
Social Functioning	87.5 (62.5–100.0)	75.0 (62.5–100.0)	87.5 (75.0–100.0)	**0.037**	0.9
Role Emotional	100.0 (66.7–100.0)	100.0 (33.3–100.0)	100.0 (66.7–100.0)	0.150	2.8
Mental Health	84.0 (68.0–92.0)	76.0 (64.0–88.0)	84.0 (72.0–92.0)	**0.006**	0.6
*PCS*	45.7 (36.7–53.5)	43.3 (35.0–50.3)	46.5 (39.1–53.8)	**0.008**	4.0
*MCS*	53.2 (44.2–58.2)	51.4 (41.6–58.0)	53.6 (45.1–58.3)	0.184	4.0

SF-36 subdomain scores and the Physical Component Summary and the Mental Component Summary are presented as median and 25th–75th percentile. *p*-values < 0.05 are depicted in bold. PCS = Physical Component Summary; MCS = Mental Component Summary.

**Table 3:** Sensitivity analysis: comparing ATAAD patient characteristics between responders and non-responders for the SF-36 questionnaire.

	Responders (*n* = 324)	Non-Responders (*n* = 231)	*p*-Value	Missing %
**Patient demographics**				
Female sex (%)	110 (34.0)	79 (34.2)	1.000	0.0
Age (median [IQR])—years	61.0 [54.0–68.0]	60.0 [51.0–67.8]	0.126	0.2
BSA (mean ± SD)—m^2^	2.00 ± 0.21	1.98 ± 0.20	0.347	30.7
History of hypertension (%)	150 (48.9)	104 (47.1)	0.749	6.0
Hyperlipidemia (%)	37 (11.7)	20 (8.8)	0.330	4.3
Diabetes mellitus (%)	3 (0.9)	6 (2.6)	0.175 ^i^	3.3
COPD (%)	14 (4.4)	11 (4.8)	0.979	3.0
Current or past smoking ≥ 1 pack years	90 (62.9)	89 (82.4)	**0.001**	58.2
History of CVA or TIA (%)	14 (4.4)	9 (4.0)	0.991	3.4
History of MI (%)	7 (2.2)	9 (4.0)	0.338	3.3
Chronic kidney disease (%)	3 (0.9)	4 (1.7)	0.458 ^i^	3.3
Prior TAA (%)	21 (6.6)	15 (6.7)	1.000	4.0
Prior aortic surgery (%)	5 (1.6)	7 (3.1)	0.375	3.3
Prior cardiac surgery (%)	13 (4.1)	8 (3.5)	0.899	2.7
Bicuspid aortic valve (%)	6 (2.1)	6 (3.0)	0.754	13.5
Known connective tissue disease (%) * No; no genetic testing performed No; genetic testing performed but not found	67 (55.4) 41 (33.9) 13 (10.7)	40 (51.3) 29 (37.2) 9 (11.5)	0.852	65.8
**Surgical procedures**				
Aortic valve surgery Ascending aortic surgery Aortic arch surgery Descending aortic surgery	210 (66.5) 315 (97.2) 225 (70.8) 2 (0.6)	116 (52.5) 226 (99.6) 148 (67.0) 2 (0.9)	**0.002** 0.053 ^i^ 0.400 1.000 ^i^	5.3 2.6 4.7 2.5
DHCA	124 (39.7)	79 (37.1)	0.602	8.1

Normally distributed continuous variables are expressed as mean ± SD, skewed continuous variables are expressed as median and 25th–75th percentile, and categorical values are expressed as percentages. *p*-values < 0.05 are depicted in bold. * Connective tissue disease diagnosed before or after acute type B aortic dissection. ^i^ = Fisher’s exact test. IQR = interquartile range; BMI = body mass index; BSA = body surface area; MI = myocardial infarction; TAA = thoracic aortic aneurysm; AAA = abdominal aortic aneurysm; DHCA = deep hypothermic circulatory arrest.

## Data Availability

Data will be shared on request to the corresponding author with permission of the disSEXion study research group.

## Supplementary Materials

The following supporting information can be downloaded at: https://www.mdpi.com/article/10.3390/jcm13082265/s1, Appendix SI: Variable definitions patient characteristics; Appendix SII: Scores of the study population compared with the general Dutch population stratified by sex; Appendix SIII: Scores of the study population compared with the general Dutch population stratified by age category; Appendix SIV: Multivariable linear regression analyses for the physical component summary (PCS) and mental component summary (MCS) scores adjusting for age and sex.

## Abbreviations

ATAAD = acute type A aortic dissection; HRQoL = health-related quality of life.

## References

[1] Evangelista A., Isselbacher E.M., Bossone E., Gleason T.G., Eusanio M.D., Sechtem U., Ehrlich M.P., Trimarchi S., Braverman A.C., Myrmel T. (2018). Insights from the International Registry of Acute Aortic Dissection: A 20-Year Experience of Collaborative Clinical Research. Circulation.

[2] Smedberg C., Steuer J., Leander K., Hultgren R. (2020). Sex differences and temporal trends in aortic dissection: A population-based study of incidence, treatment strategies, and outcome in Swedish patients during 15 years. Eur. Heart J..

[3] Hagan P.G., Nienaber C.A., Isselbacher E.M., Bruckman D., Karavite D.J., Russman P.L., Evangelista A., Fattori R., Suzuki T., Oh J.K. (2000). The International Registry of Acute Aortic Dissection (IRAD): New insights into an old disease. JAMA.

[4] Gariboldi V., Grisoli D., Kerbaul F., Giorgi R., Riberi A., Metras D., Mesana T.G., Collart F. (2007). Long-term outcomes after repaired acute type A aortic dissections. Interact. Cardiovasc. Thorac. Surg..

[5] Carbone A., Ranieri B., Castaldo R., Franzese M., Rega S., Cittadini A., Czerny M., Bossone E. (2023). Sex Differences in Type A Acute Aortic Dissection: A Systematic Review and Meta-Analysis. Eur. J. Prev. Cardiol..

[6] Eranki A., Wilson-Smith A., Williams M.L., Saxena A., Mejia R. (2022). Quality of life following surgical repair of acute type A aortic dissection: A systematic review. J. Cardiothorac. Surg..

[7] Aaronson N.K., Muller M., Cohen P.D., Essink-Bot M.L., Fekkes M., Sanderman R., Sprangers M.A., te Velde A., Verrips E. (1998). Translation, validation, and norming of the Dutch language version of the SF-36 Health Survey in community and chronic disease populations. J. Clin. Epidemiol..

[8] Thijssen C.G.E., Dekker S., Bons L.R., Gökalp A.L., Kauling R.M., van den Bosch A.E., Cuypers J., Utens E., van Kimmenade R.R.L., Takkenberg J.J.M. (2020). Health-related quality of life and lived experiences in males and females with thoracic aortic disease and their partners. Open Heart.

[9] von Elm E., Altman D.G., Egger M., Pocock S.J., Gøtzsche P.C., Vandenbroucke J.P., Initiative S. (2014). The Strengthening the Reporting of Observational Studies in Epidemiology (STROBE) Statement: Guidelines for reporting observational studies. Int. J. Surg..

[10] McHorney C.A., Ware J.E., Lu J.F., Sherbourne C.D. (1994). The MOS 36-item Short-Form Health Survey (SF-36): III. Tests of data quality, scaling assumptions, and reliability across diverse patient groups. Med. Care.

[11] McHorney C.A., Ware J.E., Raczek A.E. (1993). The MOS 36-Item Short-Form Health Survey (SF-36): II. Psychometric and clinical tests of validity in measuring physical and mental health constructs. Med. Care.

[12] Ware J.E., Sherbourne C.D. (1992). The MOS 36-item short-form health survey (SF-36). I. Conceptual framework and item selection. Med. Care.

[13] Ware J.E. (2000). SF-36 health survey update. Spine.

[14] Adam U., Habazettl H., Graefe K., Kuppe H., Wundram M., Kurz S.D. (2018). Health-related quality of life of patients after surgery for acute Type A aortic dissection. Interact. Cardiovasc. Thorac. Surg..

[15] Endlich M., Hamiko M., Gestrich C., Probst C., Mellert F., Winkler K., Welz A., Schiller W. (2016). Long-Term Outcome and Quality of Life in Aortic Type A Dissection Survivors. Thorac. Cardiovasc. Surg..

[16] Jussli-Melchers J., Panholzer B., Friedrich C., Broch O., Renner J., Schöttler J., Rahimi A., Cremer J., Schoeneich F., Haneya A. (2017). Long-term outcome and quality of life following emergency surgery for acute aortic dissection type A: A comparison between young and elderly adults. Eur. J. Cardiothorac. Surg..

[17] Immer F.F., Krähenbühl E., Immer-Bansi A.S., Berdat P.A., Kipfer B., Eckstein F.S., Saner H., Carrel T.P. (2002). Quality of life after interventions on the thoracic aorta with deep hypothermic circulatory arrest. Eur. J. Cardiothorac. Surg..

[18] Santini F., Montalbano G., Messina A., D’Onofrio A., Casali G., Viscardi F., Luciani G.B., Mazzucco A. (2006). Survival and quality of life after repair of acute type A aortic dissection in patients aged 75 years and older justify intervention. Eur. J. Cardiothorac. Surg..

[19] Sbarouni E., Georgiadou P., Manavi M., Analitis A., Beletsioti C., Niakas D., Iliodromitis E., Voudris V. (2021). Long-term outcomes and quality of life following acute type A aortic dissection. Hell. J. Cardiol..

[20] Olsson C., Franco-Cereceda A. (2013). Health-Related Quality of Life in Thoracic Aortic Disease: Part II. After Surgery on the Proximal (Root, Ascending, Arch) Aorta. Aorta.

[21] Gjeilo K.H., Stenseth R., Wahba A., Lydersen S., Klepstad P. (2018). Long-term health-related quality of life and survival after cardiac surgery: A prospective study. J. Thorac. Cardiovasc. Surg..

[22] Schachner T., Garrido F., Bonaros N., Krapf C., Dumfarth J., Grimm M. (2019). Factors limiting physical activity after acute type A aortic dissection. Wien. Klin. Wochenschr..

[23] Tang G.H., Malekan R., Yu C.J., Kai M., Lansman S.L., Spielvogel D. (2013). Surgery for acute type A aortic dissection in octogenarians is justified. J. Thorac. Cardiovasc. Surg..

[24] Bojko M.M., Suhail M., Bavaria J.E., Bueker A., Hu R.W., Harmon J., Habertheuer A., Milewski R.K., Szeto W.Y., Vallabhajosyula P. (2022). Midterm outcomes of emergency surgery for acute type A aortic dissection in octogenarians. J. Thorac. Cardiovasc. Surg..

[25] Chaddha A., Kline-Rogers E., Braverman A.C., Erickson S.R., Jackson E.A., Franklin B.A., Woznicki E.M., Jabara J.T., Montgomery D.G., Eagle K.A. (2015). Survivors of Aortic Dissection: Activity, Mental Health, and Sexual Function. Clin. Cardiol..

[26] Perk J., De Backer G., Gohlke H., Graham I., Reiner Z., Verschuren M., Albus C., Benlian P., Boysen G., Cifkova R. (2012). European Guidelines on cardiovascular disease prevention in clinical practice (version 2012): The Fifth Joint Task Force of the European Society of Cardiology and Other Societies on Cardiovascular Disease Prevention in Clinical Practice (constituted by representatives of nine societies and by invited experts). Eur. J. Prev. Cardiol..

[27] Smith J.R., Thomas R.J., Bonikowske A.R., Hammer S.M., Olson T.P. (2022). Sex Differences in Cardiac Rehabilitation Outcomes. Circ. Res..

[28] Pelosi C., Kauling R.M., Cuypers J., van den Bosch A.E., Helbing W.A., Utens E., Legerstee J.S., Roos-Hesselink J.W. (2023). Daily life and psychosocial functioning of adults with congenital heart disease: A 40–53 years after surgery follow-up study. Clin. Res. Cardiol..

[29] Pasadyn S.R., Roselli E.E., Artis A.S., Pasadyn C.L., Phelan D., Hurley K., Desai M.Y., Blackstone E.H. (2020). From Tear to Fear: Posttraumatic Stress Disorder in Patients with Acute Type A Aortic Dissection. J. Am. Heart Assoc..

[30] Ebert J.F., Huibers L., Christensen B., Christensen M.B. (2018). Paper- or Web-Based Questionnaire Invitations as a Method for Data Collection: Cross-Sectional Comparative Study of Differences in Response Rate, Completeness of Data, and Financial Cost. J. Med. Internet Res..

