# Supplementary material for: Long-Term Health-Related Quality of Life following Acute Type A Aortic Dissection with a Focus on Male–Female Differences: A Cross Sectional Study
Source: J Clin Med. 2024 Apr 13;13(8):2265. doi: 10.3390/jcm13082265 (PMC11050969; doi:10.3390/jcm13082265)
Supplement: Supplementary file 1 [file jcm-13-02265-s001.zip › jcm-2912843-supplementary.pdf]

**Appendix SI.** Variable definitions patient characteristics

| | Unit | Definition |
|---|---|---|
| *Patient demographics at ATAAD presentation* | | |
| **Age at ATAAD** | years | Age at ATAAD presentation |
| **BSA** | $m^2$ | $\sqrt{\frac{height\ (cm) x\ weight\ (kg)}{3600}}$ |
| **History of hypertension** | | Known hypertension in patient history or medical treatment for hypertension. |
| **History of hyperlipidemia** | | Medical treatment for hyperlipidemia or in patient history. |
| **Diabetes mellitus** | | Known diabetes mellitus in patient history or medical treatment for diabetes mellitus. |
| **COPD** | | Any history of Chronic Obstructive Pulmonary Disease that required medical treatment or FEV1<70%. |
| **Current or past smoking ≥ 1 pack years** | | Currently smoking or > 1 pack year in past. |
| **History of CVA or TIA** | | History of CVA or TIA |
| **History of MI** | | (N)STEMI > 90 days before presentation |
| **Chronic kidney disease** | | History of chronic kidney disease |
| **Prior TAA** | | Thoracic aortic diameter of ≥ 40 mm. |
| **Prior aortic surgery** | | Prior aortic surgery reported in patient history |
| **Prior cardiac surgery** | | Prior cardiac surgery reported in patient history |
| **Bicuspid aortic valve** | | Presence of bicuspid aortic valve, known in patient history or seen in the operation theatre |

| | |
|---|---|
| **Known connective tissue disease** | Known connective tissue disease at ATAAD presentation |

ATAAD = acute type A aortic dissection; BSA = body surface area; COPD = chronic obstructive pulmonary disease; CVA = cerebrovascular accident; MI = myocardial infarction; TAA = thoracic aortic aneurysm; DHCA = deep hypothermic circulatory arrest

**Appendix SII Scores of the study population compared with the general Dutch population stratified by sex**

| | All patients (n=324) | General population | P-value | Female (n=110) | General female population | P-value | Male (n=214) | General male population | P-value |
|---|---|---|---|---|---|---|---|---|---|
| Physical Functioning | 66.9 ± 25.6 | 83.0 ± 22.8 | <0.001 | 59.7 ± 26.2 | 80.4 ± 24.2 | <0.001 | 70.6 ± 24.6 | 85.4 ± 21.0 | <0.001 |
| Role Physical | 58.9 ± 41.7 | 76.4 ± 36.3 | <0.001 | 50.5 ± 43.7 | 73.8 ± 38.5 | <0.001 | 63.1 ± 40.1 | 78.7 ± 34.1 | <0.001 |
| Bodily pain | 79.2 ± 24.4 | 74.9 ± 23.4 | 0.002 | 76.8 ± 24.6 | 71.9 ± 23.8 | 0.039 | 80.5 ± 24.2 | 77.3 ± 22.7 | 0.056 |
| General Health | 56.2 ± 22.7 | 70.7 ± 20.7 | <0.001 | 54.0 ± 22.6 | 69.9 ± 20.6 | <0.001 | 57.4 ± 22.8 | 71.6 ± 20.6 | <0.001 |
| Vitality | 60.1 ± 24.0 | 68.6 ± 19.3 | <0.001 | 56.2 ± 24.3 | 64.3 ± 19.7 | <0.001 | 62.1 ± 23.6 | 71.9 ± 18.3 | <0.001 |
| Social Functioning | 78.6 ± 26.3 | 84.0 ± 22.4 | <0.001 | 74.3 ± 28.3 | 82.0 ± 23.5 | 0.006 | 80.8 ± 25.0 | 86.0 ± 21.1 | 0.003 |
| Role Emotional | 76.7 ± 36.3 | 82.3 ± 32.9 | 0.007 | 72.3 ± 39.0 | 78.5 ± 35.7 | 0.102 | 79.0 ± 34.7 | 85.5 ± 29.9 | 0.008 |
| Mental Health | 77.4 ± 19.6 | 76.8 ± 17.4 | 0.573 | 72.3 ± 23.2 | 73.7 ± 18.2 | 0.539 | 80.0 ± 17.0 | 79.3 ± 16.4 | 0.537 |

SF-36 subdomain scores are presented as mean ± SD in order to compare the data with the general population data which was reported as mean ± SD.
SD = standard deviation

**Appendix SIII Scores of the study population compared with the general Dutch population stratified by age category**

| | Age 41-60 | | | Age 61-70 | | | Age > 70 | | |
|---|---|---|---|---|---|---|---|---|---|
| | TAAD patients (n=73) | General population | P-value | TAAD patients (n=119) | General population | P-value | TAAD patients (n=128) | General population | P-value |
| Physical Functioning | 71.3 ± 25.2 | 84.0 ± 19.6 | <0.001 | 70.3 ± 21.9 | 71.7 ± 25.6 | 0.505 | 61.7 ± 27.8 | 58.9 ± 30.8 | 0.254 |
| Role Physical | 62.2 ± 39.1 | 74.5 ± 36.8 | 0.009 | 59.4 ± 43.2 | 67.3 ± 40.9 | 0.051 | 56.9 ± 41.6 | 56.9 ± 44.0 | 0.990 |
| Bodily pain | 80.6 ± 22.1 | 71.8 ± 24.1 | 0.001 | 79.6 ± 25.9 | 70.5 ± 24.6 | <0.001 | 78.5 ± 24.0 | 68.1 ± 27.4 | <0.001 |
| General Health | 53.7 ± 23.8 | 69.7 ± 20.6 | <0.001 | 57.2 ± 23.5 | 61.7 ± 20.2 | 0.038 | 57.1 ± 21.1 | 58.9 ± 21.1 | 0.339 |
| Vitality | 57.3 ± 23.9 | 68.6 ± 20.2 | <0.001 | 60.6 ± 24.7 | 67.7 ± 19.6 | 0.002 | 61.7 ± 22.8 | 61.8 ± 23.6 | 0.973 |
| Social Functioning | 77.4 ± 25.8 | 83.5 ± 22.1 | 0.047 | 79.2 ± 26.0 | 82.0 ± 24.6 | 0.251 | 78.9 ± 27.0 | 75.6 ± 27.0 | 0.177 |
| Role Emotional | 77.5 ± 35.5 | 81.6 ± 33.2 | 0.330 | 78.2 ± 36.5 | 81.1 ± 35.0 | 0.388 | 75.3 ± 36.5 | 74.5 ± 38.2 | 0.815 |
| Mental Health | 75.9 ± 18.2 | 75.6 ± 18.5 | 0.892 | 77.6 ± 19.9 | 76.9 ± 17.9 | 0.710 | 78.2 ± 20.3 | 73.0 ± 19.9 | 0.005 |

SF-36 subdomain scores are presented as mean ± SD in order to compare the data with the general population data which was reported as mean ± SD. P-values < 0.05 are depicted in bold. Three patients were in the age range of 16-40 year, therefore these patients were not included in the age-matched analysis.

SD = standard deviation

**Appendix SIV Multivariable linear regression analyses for the physical component summary (PCS) and mental component summary (MCS) scores adjusting for age and sex**

| | Physical component summary | | Mental component summary | |
|---|---|---|---|---|
| | Beta estimate (95% CI)* | P-value | Beta estimate (95% CI) | P-value |
| Follow-up time | -0.093 (-0.443-0.257) | 0.603 | 0.285 (-0.077-0.647) | 0.123 |
| Age at ATAAD | - | - | - | - |
| Sex (female) | - | - | - | - |
| History of hypertension | -3.428 (-5.715- -1.140) | 0.003 | -2.307 (-4.710-0.096) | 0.060 |
| History of hyperlipidaemia | -0.938 (-4.485-2.609) | 0.603 | 1.768 (-1.901-5.438) | 0.344 |
| Diabetes mellitus | 2.872 (-8.596-14.340) | 0.623 | 4.591 (-7.282-16.465) | 0.447 |
| COPD | -7.282 (-12.852- -1.713) | 0.012 | -2.701 (-8.514-3.112) | 0.361 |
| History of CVA | 2.522 (-3.364-8.308) | 0.392 | -4.305 (-10.275-1.665) | 0.157 |
| History of MI | -4.841 (-12.348-2.666) | 0.205 | 3.456 (-4.371-11.283) | 0.386 |
| Chronic kidney disease | 7.484 (-6.530-21.497) | 0.294 | 10.169 (-4.301-24.639) | 0.168 |
| Prior TAA | -4.692 (-15.912-1.824) | 0.043 | -3.016 (-7.714-1.682)_ | 0.208 |
| Prior aortic surgery | -7.044 (-15.912-1.824) | 0.119 | -0.140 (-9.407-9.126) | 0.976 |
| Prior cardiac surgery | -3.506 (-9.066-2.054) | 0.216 | -2.938 (-8.719-2.844) | 0.318 |
| BAV | 6.116 (-1.891-14.124) | -1.134 | 3.630 (-4.732-11.992) | 0.394 |
| AV surgery at ATAAD | -0.845 (-3.199-1.509) | -0.481 | 0.254 (-2.220-2.728) | 0.840 |
| Aortic arch surgery ATAAD | -1.529 (-3.973-0.915) | 0.219 | -2.379 (-4.909-0.151) | 0.065 |
| DHCA | -0.577 (-2.868-1.713) | 0.620 | -1.218 (-3.593-1.157) | 0.314 |

*Beta estimates were adjusted for age and sex in a multivariable linear regression model. Beta coefficients and corresponding 95% CI are shown. Interpretation for beta coefficients: if the beta coefficient is positive, for every unit increase in the predictor variable, the outcome variable (PCS or MCS score) will increase by the beta coefficient value.

COPD = chronic obstructive pulmonary disease; CVA = cerebrovascular accident; MI = myocardial infarction; TAA = thoracic aortic aneurysm; BAV = bicuspid aortic valve; AV = aortic valve; DHCA = deep hypothermic circulatory arrest.
